# The impact of cash transfers on social determinants of health and health inequalities in Sub-Saharan Africa: a systematic review protocol

**DOI:** 10.1186/s13643-016-0295-4

**Published:** 2016-07-13

**Authors:** Ebenezer Owusu-Addo, Andre M.N. Renzaho, Ajay S. Mahal, Ben J. Smith

**Affiliations:** Bureau of Integrated Rural Development, Kwame Nkrumah University of Science and Technology, Kumasi, Ghana; School of Social Sciences and Psychology, Western Sydney University, Sydney, Australia; School of Public Health and Preventive Medicine, Monash University, Melbourne, Australia; Nossal Institute for Global Health, University of Melbourne, Melbourne, Australia

**Keywords:** Cash transfers, Social determinants of health, Sub-Saharan Africa, Systematic review

## Abstract

**Background:**

There is increasing pressure to address the social determinants of health (SDoH) and health inequities through the implementation of culturally acceptable interventions particularly in Sub-Saharan Africa (SSA) where health outcomes are generally poor. Available evaluation research on cash transfers (CTs) suggests that the programs may influence the wider determinants of health in SSA; yet, there has been no attempt to synthesize the evidence regarding their contribution to tackling the SDoH and health inequalities. To date, nearly all the reviews on CTs' impact on health have predominantly featured evidence from Latin America with limited transferability to the social, cultural, and political environments in SSA. Therefore, the aim of this study is to undertake a systematic review to assess the role of CTs in tackling the wider determinants of health and health inequalities in SSA.

**Methods/design:**

A systematic review of published and unpublished literature on CTs’ impact on health and health determinants covering the period 2000–2016 will be undertaken. Studies will be considered for inclusion if they present quantitative or qualitative data, including all relevant study designs. The SDoH conceptual framework will be used to guide the data extraction process. EPPI Reviewer software will be used for data management and analysis. Studies included in the review will be analyzed by narrative synthesis and/or meta-analysis as appropriate for the nature of the data retrieved.

**Discussion:**

This review will provide empirical evidence on the impact of CTs on SDoH to inform CT policy, implementation, and research in SSA. The protocol follows the Preferred Reporting Items for Systematic Review and Meta-Analysis Protocols (PRISMA-P).

**Systematic review registration:**

This protocol has been registered with the PROSPERO international prospective register of systematic reviews, reference CRD42015025015.

**Electronic supplementary material:**

The online version of this article (doi:10.1186/s13643-016-0295-4) contains supplementary material, which is available to authorized users.

## Background

Western aid to Sub-Saharan African (SSA) countries has increasingly been targeted toward health [1]. This notwithstanding, the 2015 Millennium Development Goals (MDGs) report indicates that SSA’s performance across the health-related MDGs is the poorest in the world [2]. Globally, it has been estimated that there were 289,000 maternal deaths in 2013, a decline of 45 % from 1990. SSA alone accounted for 62 % (179,000) of these global deaths [3]. Similarly, although there has been a decline in under-five mortality rate, with the average annual rate of reduction increasing from 0.8 % in 1990–1995 to 4.2 % in 2005–2013, SSA still has the highest child mortality rate—92 deaths per 1000 live births, more than 15 times the average for developed countries [4]. SSA is also most hard hit by the HIV and acquired immunodeficiency syndrome (AIDS) epidemic as UNAIDS [5] estimates indicate that, in 2012, about 25 million people were living with HIV, accounting for 70 % of the global total.

Socio-economic factors such as income poverty, low level of education, poor living conditions, lack of social cohesion, limited access to water, and sanitation and poor nutrition have been identified as the key drivers of the worsening health conditions and increased health inequalities in SSA [6]. UNICEF [7] for instance, estimates that 67 % of children in SSA suffer from multiple deprivations crucial to their survival and development. The 2015 MDG report further indicates that over 40 % of the population of SSA live in extreme poverty, while the number of underweight children continues to rise in the region [2]. Clearly, efforts to improve health and reduce health inequalities in SSA need to pay greater attention to addressing the social determinants of health (SDoH) within and outside of the healthcare system.

There is a large body of evidence suggesting that tackling SDoH could lead to improved health outcomes and a reduction in health disparities [8–11]. Strategies focused on improved employment opportunities, health insurance financed by progressive taxation, and improved access to subsidized public schools are all examples of interventions targeting SDoH. A particularly promising and popular intervention that could help in this direction is cash transfer programs. Cash transfers (CTs) are generally targeted at poor households and seek to encourage increased demand for services through an “income effect” and in the case of conditional cash transfers, through both an income effect and a “substitution effect”. The potential of cash transfers to address SDoH and reduce health inequalities in SSA has not been considered in the literature. This systematic review will use the SDoH model [8] as a framework to help identify the range of health determinants upon which CTs could impact to improve health and address health inequalities in SSA. The SDoH framework provides ways of identifying the range of outcomes that CTs may achieve, and is a necessary first step to identifying or developing a theory of change. In particular, it may elucidate the hierarchy of effects that CTs achieve, and lead to the refinement of a model about the way that CTs influence SDoH and health outcomes.

### Description of the intervention

#### Cash transfers

Social protection schemes have become high on the development agendas of donors, multilateral and bilateral development agencies, and many governments in developing countries [12–15]. Social protection schemes can play three critical roles which are key to addressing the determinants of health: promotion (of livelihoods and opportunities), protection (from indulgence and human capital loss), and prevention (of poverty) [16, 17].

As a form of social assistance, CTs have become a key social protection instrument in Central Europe, Latin America, Asia, SSA, and more recently, in the USA. CTs are direct, regular, and predictable non-contributory payments that raise and smooth incomes with the objective of reducing poverty and improving household capacity to absorb financial shocks [15]. CTs have gained prominence in SSA largely due to the 2008 economic crises which compelled donors to cut aid. Rutstein [18] for instance, has argued that instead of investing large amounts in state bureaucracies, delivering small amounts of cash to poor households in SSA could be an efficient and flexible way to provide aid directly where it is needed.

Garcia and Moore [19] identify two types of CTs in SSA namely middle-income CTs (referred to as cash grants) and low-income and fragile CTs. The middle-income CTs constitute social assistance transfers which are normally located in government institutions and domestically funded with occasional donor support. These programs adopt a targeting approach to reach groups who are most in need and have no definite end-date. Low-income and fragile CTs, however, frequently have a short time frame and intend to graduate beneficiaries from the program. Low-income CTs are often seated outside government institutions, are fully or partly funded by donors, and typically target specific groups in need prioritized by donor agencies.

CT programs in SSA can be conditional cash transfers (CCTs) or unconditional cash transfers (UCTs). Both CCTs and UCTs are largely designed to transfer cash to poor households to stimulate a change in behavior. By their nature, CCTs are conditional upon beneficiary households adopting certain positive behaviors conditioned under the program, including investment in children’s education, nutrition, and healthcare in order to improve human capital and address intergenerational transmission of poverty [20]. CCTs thus constitute a kind of “social contract” that requires households to take steps to improve their lives and that of their children. The key difference between CCTs and UCTs is that the latter gives cash to households with no conditions attached [21].

The uniqueness of CTs in SSA (be it UCTs or CCTs) is that they are designed to respond to the specific challenges facing the region—such as food security and survival, as well as improving human capital. Others also focus on improving reproductive health outcomes, including STI prevention and forced or early marriage. Most programs in the region focus on supporting orphan and vulnerable children (OVC) [19].

There are marked differences in the design and delivery of CTs between SSA and other parts of the world, especially Latin America where they have been widely used. In Latin America CTs prioritize the transfer of cash to women, whereas in SSA CT programs do not specify the woman as the recipient of the transfer. CTs in SSA are also distinguished by their focus on extremely poor and labor-constrained households as well as their “soft” conditions in the case of CCTs, which are hardly monitored due to the high cost associated with tracking and enforcement [22, 23]. The diverse cultural contexts in SSA also play a key role in shaping their delivery and impact that CTs achieve. A study by Dako-Gyeke and Oduro [24] in Ghana found that though CTs were specifically targeted at vulnerable children, the Ghanaian culture whereby all children eat from the same pot resulted in the transfers being spent on all children in the household rather than those selected as direct beneficiaries. A high level of community involvement is another unique feature of CTs in SSA where, in contrast to Latin America, programs in SSA rely heavily on communities to help target the most vulnerable groups in society to receive the transfers [19]. These differences in the operation of CTs between SSA and other regions, in the nature of programs, target beneficiaries, sociocultural, ethnic, and political contexts, have implications for the impacts that may be achieved in SSA and the methods that may be used for their evaluation.

### How the intervention might work to address SDoH

**Fig. 1:**
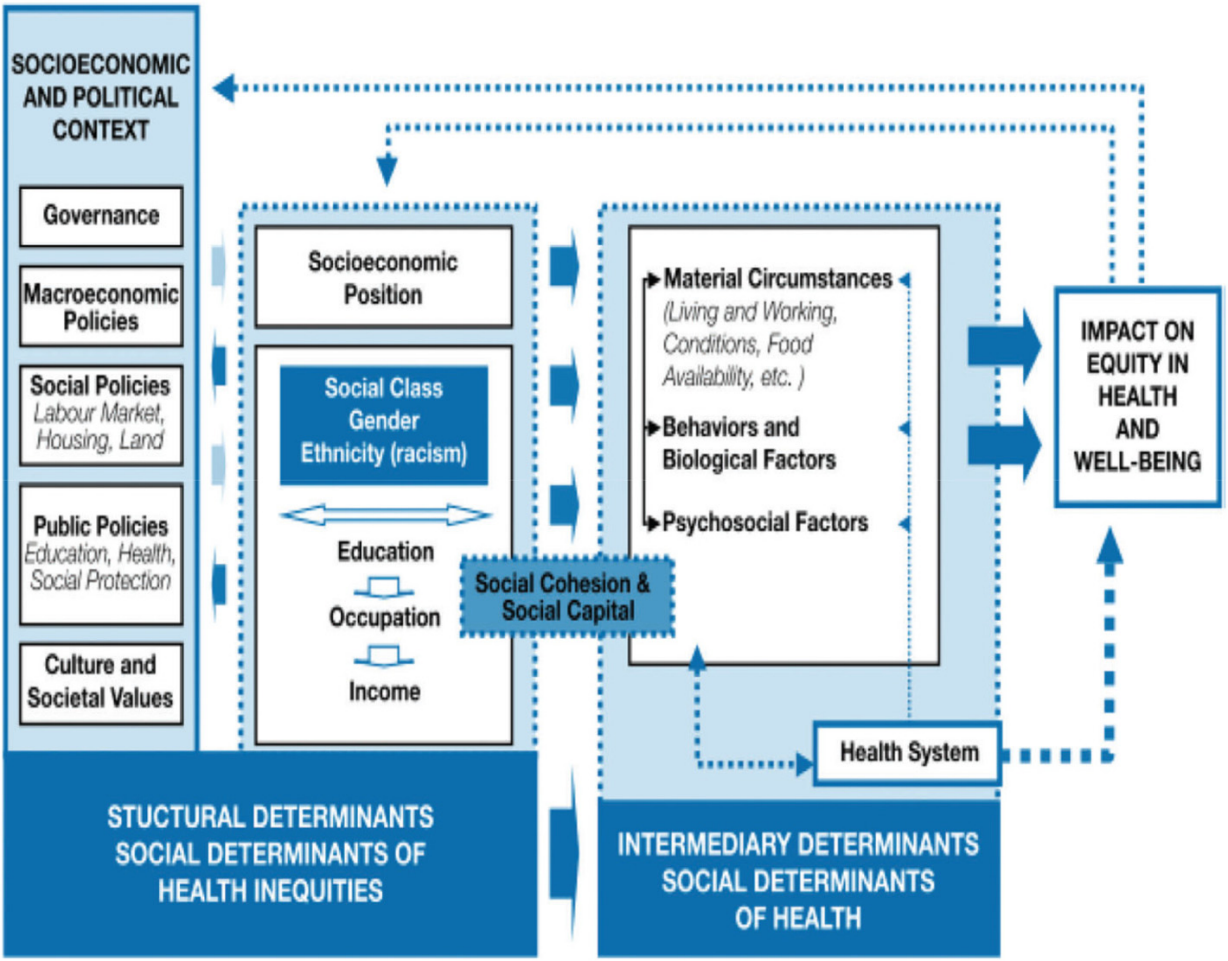
Social determinants of health framework [8], p.48

The WHO’s report titled “The Determinants of Health: The Solid Facts” concluded that biomedical interventions alone are inadequate to address the detrimental effects of poor social conditions and called for government policies which promote health equity [8]. CT programs are one example of public policies that can play a critical role in addressing the wider SDoH [25]. The SDoH conceptual framework (see Fig. 1) developed by the WHO’s Commission on Social Determinants of Health [8] shows the pathways through which socio-economic factors influence health.

Both CCTs and UCTs constitute a social policy action on the socio-economic and political context side of the SDoH framework. This review will focus on identifying the structural and intermediate SDoH upon which CTs can impact to improve health.

At the political and cultural levels, CTs may play a significant role in ensuring the participation of excluded groups in politics and traditional local hierarchies. By transferring cash to the extreme poor, CTs could strengthen the relationship between the poor and traditional authorities and disband entrenched patronage [26].

CTs emphasis on child education outcomes and enrolment could lead to improved schooling outcomes for children and potentially increased employment opportunities in adulthood, and ultimately raise socio-economic status. In the short-term, CTs can enhance household incomes which also helps improve household socio-economic position and ability to pay for health services. Thus, CTs can increase access to health services by less well-off groups [27], which in turn, may result in improved health outcomes.

CTs can also impact on intermediary determinants of health by directly addressing elements of material deprivation. For instance, cash transfers may increase household income and capacity to purchase more nutritious food, clothes and other basic needs. Increases in household income can similarly result in improvements in psychosocial circumstances such as relief from worries regarding meeting basic needs and stressful living conditions, and ultimately improve mental health and well-being [28].

In addition to the above, CTs may affect social capital and improve social cohesion at the individual and community levels. Some CTs require women to attend nutrition education meetings and this could afford women the opportunity to interact frequently and network with their peers—thus increasing linking social capital which could improve health outcomes [29]. In sum, there are a number of ways by which CTs can tackle the structural and intermediate social determinants of health to potentially impact on equity in health and well-being.

### Why it is important to do this review

Available evaluation research on CTs in SSA suggests that the programs may influence the wider determinants of health, yet there has been no attempt to synthesize the evidence regarding their contribution to tackling the SDoH. To date, reviews of the impact of CTs have tended to focus on health services and health outcomes [27, 30–38], HIV [39, 40], nutrition [41, 42], and other social outcomes such as education [21] and child labor [43, 44]. These reviews have left a gap in our knowledge, particularly about the impact of CTs upon a wider range of health determinants and their effect on health inequalities. This knowledge is required to guide the further development and adaptation of CTs to effectively address the SDoH and reduce health inequalities in SSA. Moreover, existing reviews of the effect of CTs have relied heavily on evidence from Latin America, with limited contextual and programmatic relevance to SSA. Finally, none of the previous reviews have included qualitative evidence on the programs’ impacts (including the unintended, unanticipated impacts) on health and how the program works to improve health. The lack of inclusion of qualitative evidence in these previous reviews has resulted in a limited understanding of the operation and range of impacts of CTs in different contexts.

This systematic review aims to address the above gaps by synthesizing quantitative and qualitative evidence on the contribution of CTs in addressing the wider SDoH and health inequalities in SSA. As of 2012, there were about 123 CT programs being implemented in SSA with rigorous evaluations [19]. This review will add to the limited body of knowledge on the role of multi-dimensional interventions in improving health and its determinants in SSA [8, 45].

### The review question

The aim of the review is to conduct an evidence synthesis of the impact of CTs in tackling the SDoH and health inequalities in SSA. To achieve the aim of the review, three review questions (RQs) have been formulated:

- RQ1. What are the effects of cash transfer programs on social determinants of health in Sub-Saharan Africa?
- RQ2. What are the effects of cash transfer programs on health outcomes for different populations in SSA?
- RQ3. What are the barriers and facilitators of a successful CT intervention for improved health outcomes?

## Methods/design

Traditionally, the methodological criteria used to include studies in systematic reviews of effectiveness have tended to focus on studies with designs that yield an unbiased estimate of interventions effects. In this regard, randomized controlled trials (RCTs) are prioritized as the “gold standard” and to some extent quasi-experiments. However, relying solely on RCTs and other quasi-experimental designs is likely to be insufficient to fully assess policy interventions, including health promotion activities and CTs [46, 47]. As with health promotion interventions, CTs tend to include broadly defined activities that are best evaluated using a wide variety of study designs [22, 47]. Davis et al. [22] observe that CTs in SSA have been evaluated using RCTs, quasi-experiments as well as qualitative methods [22]. Recently, Petticrew [48] has argued for the inclusion of both quantitative and qualitative assessments in systematic reviews of social policy interventions. He notes that while quantitative studies are useful in determining the effects of interventions, qualitative evidence helps to clarify the range and nature of impacts, and which groups experience these impacts. Qualitative evidence can also help better understand the barriers and facilitators of effective CT interventions and their acceptability, as well as outcomes that cannot be readily measured quantitatively [48, 49].

As a good practice and due to the complexities associated with doing a systematic review of this nature, to minimize methodological problems (validity and bias) [50], validated guidelines from the Cochrane Collaboration will be adapted in carrying out the review. The protocol will adhere to the Preferred Reporting Items for Systematic Reviews and Meta-Analyses Protocols reporting guidelines (PRISMA-P) [51]. A PRISMA-P checklist is included with this manuscript (see additional file 1).

### Inclusion criteria

#### Types of studies

To address RQ1 and RQ2, both quantitative and qualitative evidence will be considered. For quantitative evidence, studies that investigate the effects of CTs using experimental and quasi-experimental study designs will be included. Specifically, studies using the following study designs will be examined:

- randomized and cluster-randomized controlled trials (RCTs, c-RCTs);
- regression discontinuity designs (RDDs);
- interrupted time series (ITS) (with at least one time-point before and one after the intervention and a clearly defined intervention point);
- controlled before-after studies (CBAs) with outcomes assessed at the same time points in the intervention and the control group, and comparable intervention and control sites; and
- cross-sectional studies with pre-and post-test measures of the outcome variables of interest and using appropriate methods to match participants and non-participants (propensity score or covariate matching) or statistical methods to control for selection bias and confounding (e.g., difference-in-differences, and single difference regression analysis, Heckman selection models and instrumental variables).

Aside from quantitative studies that measure impacts of CT interventions, stand-alone qualitative studies and qualitative data embedded in quantitative studies (e.g., focus groups, interviews, case studies, participatory action research, observation) that have identified CTs’ impacts and/or perceptions of beneficiaries and stakeholders concerning the range of impacts resulting from CTs, will be considered for RQ1 and RQ2 to complement the quantitative findings. The use of qualitative studies here is to provide insights into a broader range of impacts of CTs upon the SDoH and health inequalities.

To address RQ 3, the following study designs will be included:

- stand-alone qualitative studies and qualitative studies conducted together with quantitative studies (e.g., focus groups, interviews, case studies, participatory action research, observation), and;
- process evaluations, providing contextual and other important influences upon CT delivery (not only effects), which elucidate barriers and facilitators.

### Types of participants

The analysis will be restricted to countries in SSA. The population of focus in this study is those targeted by either CCT or UCT programs. CT programs in SSA target OVC, HIV-affected individuals, the elderly, and people with disabilities or those who are unable to participate in the labor market [19].

### Types of interventions

The review will include CCTs and UCTs which aim to reduce poverty meeting the following criteria:

- consists of direct CTs to households or individuals;
- non-contributory, that is, the cash transfer is not a payment from a social insurance system that recipients have previously contributed to;
- the household or individual is the recipient of the transfer, with CTs directed to whole communities excluded;
- provided by a formal institution (state/governmental, international or non-governmental organization), and;
- has a goal of reducing income poverty, or is targeted to specific populations perceived to be at increased health or financial risk (e.g., HIV infection, old age, or disability).

All intervention variations in terms of size of the CT, frequency of the transfer, beneficiary of the transfer, and mode of delivery will be included. UCT programs that are stand-alone will be included but those delivered in conjunction with or alongside other interventions will be excluded. CTs for assistance in humanitarian disasters will be excluded as they are often one-time and address different causal pathways.

Two comparators will be included to assess CTs impact on health and the wider determinants of health: studies that compare CTs (either CCTs or UCTs) with control groups or non-CT comparison groups that receive normal services, and those that make comparison between a CCT intervention and a UCT intervention.

### Types of outcomes

An initial scoping of the literature suggests that there will be considerable heterogeneity in both primary and intermediate health and SDoH outcome measures used in evaluations of CT interventions. Therefore, for the purpose of this review, outcomes will not be specified priori, although they may include the following:

#### Primary outcomes

As CT programs have multiple components, they have the potential to impact a broad range of socio-economic outcomes which are intrinsically linked to health and well-being. Therefore, studies will be included in the review if they report at least one of the socio-economic outcomes listed below.

- Financial poverty:
  - o household income—measured by consumption expenditure or income levels;
  - o poverty—incidence or prevalence of households below an income (or expenditure) threshold, or;
  - o household assets.
- Employment/occupation.
- Education and literacy—enrolment, drop out, grade promotion.
- Individual or community empowerment.
- Enhanced social inclusion, cohesion, or capital.
- Child labor.
- Civic participation, shared decision making, or demand for accountability from duty bearers.
- Living conditions (e.g., housing, sanitation).
- Neighborhood conditions (e.g., level of crime and violence) and services.

### Secondary outcomes

Health and quality of life, the secondary outcomes for this review relating to health inequalities are:

- Health-related quality of life—examples being mental well-being, life satisfaction or happiness, and physical well-being;
- sexual health behaviors—for example contraceptive use, changes in sexual behavior or outcomes;
- access to health care— such as having health insurance, utilization of health services, increased access for disadvantaged groups or a reduction in gaps in coverage;
- changes in health outcomes, measured by morbidity, mortality, child development, and HIV prevalence or incidence, and;
- nutrition—food consumption/security and anthropometry measures.

### Search methods

Due to resource constraints for translation, the search will be limited to studies published in English. It will also be limited to studies published after 2000 since the majority of CTs in SSA started around this time [19]. Due to the poor publication culture in SSA [52], and to avoid selection bias [53] the search will include published and unpublished studies, including refereed and non-refereed journals, conference proceedings, book chapters, working papers, dissertations, government reports, non-governmental reports, and other technical reports. Published comments, expert opinions, editorials, book reviews, op-eds, summaries, or media briefings will be excluded.

A comprehensive search for studies will be performed across a broad range of information sources to reflect the multidisciplinary nature of the topic and to avoid reporting bias [50].

### Electronic searches

#### Databases

Additional file 2 presents the initial search strategy for Ovid MEDLINE performed on 30 July 2015. This search strategy will be used to search the following databases for relevant records:

#### Health and biomedical

- Cochrane Central Register of Controlled Trials (CENTRAL) (*The Cochrane Library*)
- Cochrane Public Health Group Specialized Register
- The Campbell Library: The Campbell Collaboration
- Ovid MEDLINE
- EMBASE
- CINAHL
- PsycINFO
- PubMed
- ScienceDirect
- African Healthline
- Wiley Online Library
- EPOC Register

### Social Science

- Social Sciences Citation Index
- Sociological Abstracts
- Applied Social Sciences Index and Abstracts (ASSIA)
- Web of Science
- Social Science Research Network—SSRN eLibrary

### Business and Economics

- Business Source Complete
- EconLit

### Multidisciplinary

- Scopus (1995 to present)
- Academic OneFile

The subject heading terminology and syntax of search terms will be adapted according to the requirements of the individual databases.

### Journals to be hand-searched

- Journal of Development Economics
- Journal of Development Effectiveness
- Health Policy and Planning
- Global Health

Reference lists from included studies will be scanned for further relevant articles and study authors; individual experts and organizations will be contacted to obtain relevant published, unpublished, or ongoing studies.

### Gray literature

The following websites will be searched for reports and unpublished papers.

- 3ie impact database
- ProQuest Dissertations & Theses Database
- The Directory of Open Access Repositories—OpenDOAR (www.opendoar.org/)
- EconPapers (www.econpapers.repec.org)
- Social Science Research Network—SSRN eLibrary (www.ssrn.com/)
- Transfer Project (http://www.cpc.unc.edu/projects/transfer)—a project focusing on evaluation of cash transfers in SSA
- World Bank (www.worldbank.org)
- UNICEF (www.unicef-irc.org/)
- USAID Database
- African Development Bank (www.afdb.org)
- Asian Development Bank (www.adb.org)
- European Bank for Reconstruction and Development (www.ebrd.com)
- Inter-American Development Bank (www.iadb.org)
- WHO (www.who.int/library/databases/en/)
- United Kingdom Department for International Development (www.gov.uk/government/organisations/department-for-international-development)
- IDEAS Repec (https://ideas.repec.org/)
- IPA
- J-PAL

### Internet search engines

Google Scholar and Scirus Internet search engines will be searched using terms similar to those used for searches of the bibliographic databases.

Websites and online resources of universities and research centers in Africa will be searched for working papers and dissertations.

Throughout the search process, a “search log” will be kept in order to record the searching process. This is to ensure that the review methods are transparent and facilitate judgment about the quality of the results [52].

### Data collection and analysis

#### Selection of studies

All relevant records will be downloaded into the review management software EPPI reviewer. An inclusion criteria worksheet will be prepared, and each reference screened. At the initial stage, titles and abstracts will be scanned to exclude duplicate records and remove those clearly outside the scope of the review. Full-text papers potentially meeting the inclusion criteria based on content of titles and abstracts will be retrieved. Multiple publications and reports will be linked. One independent author will screen the full papers to determine eligibility for inclusion and will consult a second independent author to build consensus and resolve any disagreements that may arise. Reasons for exclusion of papers will be recorded.

#### Data extraction and management

The data extraction form recommended by the Cochrane Public Health Group “Guide for Developing a Cochrane Protocol” [54] will be adapted for the extraction of data from quantitative studies addressing RQ1 and RQ2. A draft quantitative data extraction form is shown in Additional file 3. The quantitative data extraction form includes the following categories: study characteristics (country, aim of study), socio-demographic characteristics of participants (education, gender, age, occupation, socio-economic status), characteristics of intervention (type of CT, aims of CT, duration of intervention, target beneficiaries, intended outcome), methods (study design, study population and sampling, data collection), outcomes and results of the study, comments about the study, and authors conclusions. The Joana Briggs Institute’s [55] data extraction form for qualitative studies will be adapted for extracting data from qualitative and process evaluation studies. The qualitative data extraction form is shown in Additional file 4. Data extraction forms will be piloted (using representative samples of the included studies) and refined so as to reduce bias and improve the validity and reliability of the review [52].

The conceptual framework presented in Fig. 1 will be used to facilitate categorization of studies. EPPI Reviewer 4 will be used to aid the data management and analysis. Adverse findings reported quantitatively or qualitatively will be recorded. If key information is missing from reports, or clarifications are required, authors will be contacted to obtain the information.

#### Risk of bias/quality assessment

**Fig. 2:**
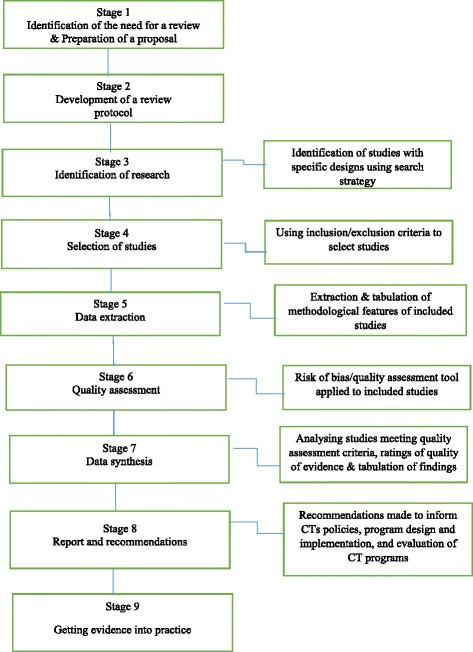
Summary of quality assessment at various stages of the review

Given the huge impact that poor quality assessment has on the results of systematic reviews, methodological quality of studies and generalizability/transferability of findings will be assessed at a number of stages of the review process (see Fig. 2). The critical appraisal tool developed by Cochrane Effective Practice and Organization of Care (EPOC) [56] will be used to assess risk of bias for randomized controlled trials, quasi-randomized trials, controlled before-after studies, and interrupted time-series studies. The Quality Assessment Tool for Quantitative Studies developed by the Effective Public Health Practice Project [57] will be used to appraise all other quantitative studies. Additional file 5 indicates the risk of bias assessment criteria. In the case of qualitative and process evaluation studies, the critical appraisal will be done using the Joana Briggs Institute’s [55] Qualitative Assessment and Review Instrument (JBI-QARI). The JBI-QARI tool has been identified as the most coherent appraisal tool for qualitative research [58]. Additional file 6 indicates the criteria for the critical appraisal of qualitative studies.

Tables will be used to summarize the internal validity of studies included in the review. Quantitative studies will be appraised as having a high, low, or unclear risk of bias. The methodological quality of qualitative studies will be assessed for dependability. The validity assessment forms will be piloted in the same way as the data extraction form. One reviewer will independently carry out the risk/quality assessment with a second reviewer checking the assessment results. When disagreement arises, a third review will be used to build consensus.

#### Measures of treatment effects

For quantitative studies addressing RQ1 and RQ2, it is anticipated existing studies will have used a variety of instruments and different analytical approaches to measure the impact of CTs. Where possible, the necessary data will be extracted to calculate standardized effect sizes. For dichotomous outcomes, an odds ratio will be calculated as the measure of effect. For continuous outcomes, standardized means difference (Hedges’ *g*) will be computed to limit small sample bias. For regression-based studies reporting correlation coefficients, Fisher’s *z* will be computed. All calculations will be based on the formulae provided by Borenstein et al. [59].

To facilitate a meta-analysis, the measures of effect sizes will be standardized for studies judged to be comparable in order to obtain a uniform scale. The standardized mean difference (in this case Hedges’ *g*) will be used in order to compare and combine results of continuous data, binary data, and correlation data. Using the methods described by Borenstein et al. [59], log odds ratios and Fisher’s *z* will be transformed to standardized mean difference (Hedges’ *g*) to obtain a common matric for the meta-analysis. Effect sizes, standard errors, and their 95 % confidence intervals will be computed as recommended by Borenstein et al. [59]. In situations where the required data cannot be extracted, authors will be contacted through email or phone for the information to calculate the effect size. If all efforts to establish the standardized effect sizes are unsuccessful, then the measure of treatment effect that is provided in the primary research will be reported in a narrative synthesis.

#### Dependent effect sizes

It is a requirement that the effect sizes included in a meta-analysis model are independent [59]. However, if meta-analysis is possible in this review, it is anticipated that there will be issues of dependent effect sizes from a range of sources including studies using a single control group and more than one treatment group, more than one study being reported in a single paper, a number of papers reporting findings of one study, studies measuring outcomes at more than one point in time, and studies reporting multiple outcomes.

To overcome this, a number of steps will be taken to ensure that only independent findings are included in meta-analysis. Where studies use a single control group and more than one treatment group (in this case non-CT comparison group versus a CCT intervention and a UCT intervention), the aim will be to compute a summary effect for CCTs versus control and UCTs versus control and include in separate meta-analyses in line with the treatment construct. In situations where a large number of included studies have the same treatment groups (in this case CCTs and UCTs), which allows for the computation of the same effect size (CCTs versus UCTs), a direct comparison of CCTs and UCTs will be performed, ignoring the control group (non-CT comparison group) as recommended by Borenstein et al. [59].

Where multiple papers are identified on a single study, the one considered to be the “main” paper (the one with the most relevant data) will be selected and the others will be consulted for additional information if necessary. As it is possible that primary studies can produce independent effect sizes within a single study report, where a single paper clearly describes more than one study the effect sizes will be treated as independent. As indicated on the data extraction forms, information will be collected about funding bodies and program names to help identify studies which might be linked or split. For included studies which report multiple outcome measures, the outcome that is most commonly reported across included studies or the outcome that is most accurately measured will be used. Only one effect estimate per study will be included in a single meta-analysis. For included studies reporting follow-up effects at multiple points in time, the final follow-up measure will be used to determine the effect of the intervention.

#### Unit of analysis

For this review, the unit of analysis may be individual, household, school, health facility, community or other cluster. There will be no restrictions to studies with a particular unit of analysis. For cluster-randomized trials, the intention is to ensure that there is adjustment for the design effect that may arise from group-based allocation. If unit of analysis errors exist, this will be corrected by adjusting the standard errors or sample sizes from cluster-randomized trials using the methods described in the Cochrane Handbook for Systematic Reviews of Interventions [50]. Individual level data will be collected from study authors by email to aid the necessary cluster adjustments. Included studies with unit of analysis issues for which individual level data for cluster adjustment cannot be retrieved will be excluded from the meta-analyses.

#### Dealing with missing data

Study authors will be contacted through email when study designs, methods or outcomes are unclear or have not been reported. When no success is achieved in obtaining missing data, this will be indicated in the narrative description of the study. The quantity of missing data will be considered in the review and will discuss the potential impact on the findings and conclusions.

### Data synthesis

#### Quantitative analysis and synthesis

In synthesizing the quantitative evidence regarding RQ1 and RQ2, meta-analysis will be considered for studies (e.g., RCTs, c-RCTs, RDDs, CBAs, and ITS) with the same outcome if they are comparable across population, intervention, comparator, and outcome (PICO) elements. This will be performed with EPPI Reviewer 4 using a random-effects model to address heterogeneity. Meta-analysis will only be conducted for studies which are assessed to be sufficiently similar. Therefore, where effect sizes cannot be pooled due to extreme heterogeneity, a forest plot will be used to illustrate the range of effect sizes. For included studies where no standardized effect size can be obtained for meta-analysis, effect sizes, and confidence intervals will still be reported but not included in the meta-analysis. Results of experimental and quasi-experimental studies will be analyzed separately.

Where possible, sensitivity analysis will be conducted to assess the sensitivity of results to quality of data and the approach to analysis. The scope of the sensitivity analysis will include a re-run meta-analysis with only studies assessed to be of a low risk of bias.

If meta-analysis is not possible due to heterogeneity in studies, narrative synthesis will be used to synthesize quantitative evidence regarding RQ1 and RQ2, following the approach recommended by Popay et al. [60]. The narrative synthesis steps will include developing a theory of how CTs impact upon the SDoH and health inequalities (see Fig. 1), undertaking a preliminary synthesis of the findings, exploring the relationships in the data, and assessing the robustness of the synthesis. The data from the included studies will be used to provide a textual and a visual summary of the results, grouping findings by the type of CT intervention, study characteristics, study participants, setting/context, outcomes measured, and their respective effect sizes. To identify the factors that might account for the differences in direction and size of effects across included studies, relationships will be explored within and between similar studies paying particular attention to characteristics of individual studies and their reported findings and the findings of different studies.

Whether or not a meta-analysis is possible, a summary of findings table will be used to present the findings for each primary outcome. One review author will organize the individual study findings into broad descriptive domains (study outcomes) which will be presented in summary tables accompanied by brief descriptions of the study findings associated with the outcome domain. A second author will then review the narrative description of the studies and check that the domains in the summary table are appropriate. Drawing on the approach described by Ogilvie [61], harvest plots will be used to visually convey findings relating to the effects of CTs on health inequalities along PROGRESS categories [50]. In line with the Cochrane Public Health Group [54] guidelines, the Grading of Recommendations Assessment, Development and Evaluation (GRADE) approach [62] will be used to assess the quality of body of evidence from all quantitative studies within each broad outcome domain. Following GRADE, the quality of the body of evidence for each study outcome will be judged as either: high quality, moderate quality, low quality or very low quality.

#### Assessment of heterogeneity

It is possible that there may be substantial differences in populations, interventions and because outcomes of interest will vary across studies, which could preclude meta-analysis. Study heterogeneity will first be examined based on sound scientific judgment to determine the degree of similarity and differences in interventions, participants or outcomes. In the event that a meta-analysis is appropriate, statistical heterogeneity of effects will be assessed using chi-square and I-square. Efforts will be made to analyze the factors explaining heterogeneity through moderator analysis, including sub-group meta-analysis.

#### Moderator analyses

Provided that suitable data are available, moderator analysis will be conducted to explore sources of heterogeneity. Moderator analysis will be performed on methodological variables (study design, risk of bias/study quality, and length of follow-up) to examine their potential influence on reported outcomes. If the included studies report effect sizes disaggregated by characteristics of CTs’ recipients, sub-group analyses will be conducted to investigate heterogeneity of the outcomes based on the following characteristics: age (children and adults), gender (female and male), level of education, level of household income, and location (urban and rural). If the sub-group analyses include a large number of studies to conduct meaningful statistical testing, *t* tests and chi-square tests will be calculated to determine the statistical significance of sub-group differences in treatment effects.

#### Assessment of publication biases

Aside from the use of a comprehensive search strategy and the inclusion of a variety of study designs to deal with publication bias, if more than 10 eligible studies reporting the same outcome are identified, funnel plots will be used to assess reporting bias.

#### Qualitative analysis and synthesis

For RQ1 and RQ2, a description of qualitative studies will be performed to give insights into the identified impacts of CTs upon SDoH and health inequalities. A similar approach will be used to address RQ3. A thematic synthesis of qualitative studies will be carried out to combine the evidence using the approach proposed by Thomas and Harden [63]. A deductive approach will be used to develop themes emanating from the data in line with the SDoH framework (Fig. 1). For qualitative studies included in this review, the ConQual approach developed by Munn et al. [64] will be used to assess the findings based on dependability and credibility as either high quality, moderate quality, low quality, or very low quality. ConQual is similar to GRADE in that both assess the degree of confidence that can be reposed in synthesized findings [65]. Themes identified in the qualitative and/or process evaluation studies will be used to complement and/or interpret the findings of the quantitative studies as part of the narrative synthesis, and recommendations will be drawn for CT program design and implementation in SSA.

### Applicability and transferability

Applicability and transferability are essential components of interventions like CTs and will be discussed in this review. Armstrong et al. [47] recommends that applicability and transferability should be considered in health promotion and public health reviews so as to translate the findings of a review to a given population, intervention, or setting. While in this review applicability will not be explicitly investigated, factors relating to process, implementation, and local context will be collected and reported on.

## Discussion

There is increasing pressure to address the SDoH and health inequities through the implementation of culturally acceptable interventions, particularly in SSA. This systematic review aims to add to the extant literature by synthesizing the evidence on the impact of a widely used social assistance program (CTs) on the wider determinants of health. The findings will need to be considered alongside the complexities associated with systematic reviews in public health and health promotion. The evidence synthesis provided by the review will contribute to CTs policy, implementation, and research in SSA.

One of the major strengths of this review is the use of a systematic and transparent approach, employing validated and recommended tools and methods. The main limitation, however, is that searching for studies on SDoH and/or health inequalities is difficult due to the range of disciplines involved in this research (e.g., public health, economics, anthropology), and the searches can also be affected by lack of sensitivity (breadth of coverage) and specificity (efficiency of searching) [47]. To overcome this challenge, the search for papers in this review covers a wide range of sources and the search strategy will also be piloted and revised.

### Dissemination plans

This systematic review will form a chapter of EOA’s PhD. The results will also be disseminated through peer-reviewed publication, conference, and formal presentation. Though at present there are no plans for updating the review, this will be considered if a significant amount of new data becomes available.

### Study registration

This systematic review has been registered with PROSPERO—the International Prospective Register of Systematic Reviews, registration number: CRD42015025015.

## Abbreviations

AIDS, acquired immunodeficiency syndrome; ASSIA, Applied Social Sciences Index and Abstracts; CBAs, controlled before-after studies; CCTs, conditional cash transfers; CENTRAL, Cochrane Central Register of Controlled Trials; CSDH, Commission on Social Determinants of Health; CTs, cash transfers; c-RCTs, cluster-randomized controlled trials; EPOC, effective practice and organization of care; HIV, human immunodeficiency virus; ITS, interrupted time series; JBI, Joana Briggs Institute; GRADE, Grading of Recommendations Assessment, Development and Evaluation; PICO, population, intervention, comparator and outcome; PRISMA-P, Preferred Reporting Items for Systematic Review and Meta-analysis Protocols; PROGRESS, place of residence, race or ethnic origin, occupation, gender, religion, education, socio-economic status, and social capital; PROSPERO, International Prospective Register of Systematic Reviews; SSA, Sub-Sahara Africa; EPHPP, Effective Public Health Practice Project; QARI, Qualitative Assessment and Review Instrument; RCTs, randomized controlled trials; RQs, review questions; SDoH, social determinants of health; UCTs, unconditional cash transfers; UNAIDS, Joint United Nations Programme on HIV and AIDS; UNICEF, United Nations International Children’s Emergency Fund; WHO, World Health Organization.

## Additional files

Additional file 1: PRISMA-P checklist. (PDF 136 kb) Additional file 2: Sample search strategy. (PDF 163 kb) Additional file 3: Data extraction form—quantitative studies. (PDF 572 kb) Additional file 4: Data extraction form—qualitative studies. (PDF 536 kb) Additional file 5: Risk of bias assessment. (PDF 158 kb) Additional file 6: Critical appraisal of qualitative studies. (PDF 150 kb)
