# Supplementary material for: The impact of cash transfers on social determinants of health and health inequalities in Sub-Saharan Africa: a systematic review protocol
Source: Syst Rev. 2016 Jul 13;5:114. doi: 10.1186/s13643-016-0295-4 (PMC4944314; doi:10.1186/s13643-016-0295-4)
Supplement: Additional file 1: — PRISMA-P checklist. (PDF 136 kb) [file 13643_2016_295_MOESM1_ESM.pdf]

PRISMA-P Checklist

Section/topic	Item #	Checklist item	Reported on page #
**ADMINISTRATIVE INFORMATION**			
**Title**			
Identification	1a	Identify the report as a protocol of a systematic review	Page 1
Update	1b	If the protocol is for an update of a previous systematic review, identify as such	N/A
Registration	2	If registered, provide the name of the registry (e.g., PROSPERO) and registration number	PROSPERO 2015:CRD42015025015
**Authors**			
Contact	3a	Provide name, institutional affiliation, and e-mail address of all protocol authors; provide physical mailing address of corresponding author	Page 1
Contributions	3b	Describe contributions of protocol authors and identify the guarantor of the review	Page 23
Amendments	4	If the protocol represents an amendment of a previously completed or published protocol, identify as such and list changes; otherwise, state plan for documenting important protocol amendments	N/A
**Support**			
Sources	5a	Indicate sources of financial or other support for the review	N/A
Sponsor	5b	Provide name for the review funder and/or sponsor	N/A
Role of sponsor/funder		Describe roles of funder(s), sponsor(s), and/or institution(s), if any, in developing the protocol	N/A
**INTRODUCTION**			
Rationale	6	Describe the rationale for the review in the context of what is already known.	Page 7
Objectives	7	Provide an explicit statement of the question(s) the review will address with reference to participants, interventions, comparators, and outcomes (PICO)	Page 8
**METHODS**			
Eligibility criteria	8	Specify the study characteristics (e.g., PICO, study design, setting, time frame) and report characteristics (e.g., years considered, language, publication status) to be used as criteria for eligibility for the review	Pages 9-11

PRISMA-P Checklist

Information sources	9	Describe all intended information sources (e.g., electronic databases, contact with study authors, trial registers, or other grey literature sources) with planned dates of coverage	Pages 12-14
Search strategy	10	Present draft of search strategy to be used for at least one electronic database, including planned limits, such that it could be repeated	Additional file 2
Study Records			
Data management	11a	Describe the mechanism(s) that will be used to manage records and data throughout the review.	Page 15
Selection process	11b	State the process that will be used for selecting studies (e.g., two independent reviewers) through each phase of the review (i.e., screening, eligibility, and inclusion in meta-analysis)	Page 15
Data collection process	11c	Describe planned method of extracting data from reports (e.g., piloting forms, done independently, in duplicate), any processes for obtaining and confirming data from investigators	Page 15
Data items	12	List and define all variables for which data will be sought (e.g., PICO items, funding sources), any pre-planned data assumptions and simplifications	Pages 10-12
Outcomes and prioritization	13	List and define all outcomes for which data will be sought, including prioritization of main and additional outcomes, with rationale	Page 10-12
Risk of bias in individual studies	14	Describe anticipated methods for assessing risk of bias of individual studies, including whether this will be done at the outcome or study level, or both; state how this information will be used in data synthesis.	Page 16
**Data**			
Synthesis	15a	Describe criteria under which study data will be quantitatively synthesized	Page 16-18
	15b	If data are appropriate for quantitative synthesis, describe planned summary measures, methods of handling data, and methods of combining data from studies, including any planned exploration of consistency (e.g., I^2, Kendall's tau	Page 16-18
	15c	Describe any proposed additional analyses (e.g., sensitivity or subgroup analyses, meta-regression)	Page 20
	15d	If quantitative synthesis is not appropriate, describe the type of summary planned	Page 19
Meta-bias (es)	16	Specify any planned assessment of meta-bias(es) (e.g., publication bias across studies, selective reporting within studies)	Page 18
Confidence in cumulative evidence	17	Describe how the strength of the body of evidence will be assessed (e.g., GRADE)	Page 19

PRISMA-P Preferred Reporting Items for Systematic review and Meta-Analysis Protocols.
