# Supplementary material for: The impact of cash transfers on social determinants of health and health inequalities in Sub-Saharan Africa: a systematic review protocol
Source: Syst Rev. 2016 Jul 13;5:114. doi: 10.1186/s13643-016-0295-4 (PMC4944314; doi:10.1186/s13643-016-0295-4)
Supplement: Additional file 2: — Sample search strategy. (PDF 163 kb) [file 13643_2016_295_MOESM2_ESM.pdf]

Additional file 1: Search strategy for Ovid MEDLINE(R) 1946 to Present with Daily Update

#	Searches
1	Social Welfare/
2	exp Social Security/
3	Maternal Welfare/ or Public Assistance/
4	Public Policy/
5	(social adj (assistance or polic* or welfare or insurance* or protection)).tw.
6	((financial or cash or pay* or monetary or money or monies) adj2 (transfer* or measure* or incentive* or allowance* or exclu* or reform* or gain* or credit*1 or benefit*1)).tw.
7	((saftety net* or anti-poverty) adj2 (program* or intervention*)).tw.
8	((family or families) adj2 (polic* or program* or intervention*)).tw.
9	(allowance* adj2 (intervention* or program* or assistance*)).tw.
**10**	or/1-9
11	(sub-saharan africa or east* africa or west* africa or southern africa or central africa or north* africa).sh,tw.
12	(((Angola* or Benin or Botswana* or Burkina Faso or Burundi* or Cameroon* or Cape Verde* or Central African Republic or Chad* or Comoro* or Democratic Republic of the Congo or Congo* or Cote d'Ivoire or Djibouti or Equatorial Guinea* or Guinea-Bissau or Eritrea* or Ethiopia* or Gabon* or Gambia* or Ghana* or Guinea* or Kenya* or Lesotho or Liberia* or Madagascar or Malagasy or Malawi* or Mali* or Mauritania* or Mauritius or Mozambi* or Namibia* or Niger* or Nigeria* or Rwanda* or Sao Tome) and Principe) or Senegal* or Seychelles* or Sierra Leone* or Somalia* or South Africa* or Swaziland* or Sudan* or Tanzania* or Western Sahara or Togo* or Uganda* or Zambia* or Zimbabwe*).sh,tw.
**13**	**11 or 12**
14	(randomized controlled trial or controlled clinical trial).tw.
15	(random* or trial or placebo).tw.
16	random allocation/
17	single-blind method/ or double-blind method/
18	((single or double or triple or treble) adj blind*).tw.
19	Longitudinal studies/ or Retrospective studies/ or Follow-up studies/
20	Intervention studies/ or Cross-over studies/ or Cross-sectional studies/
21	Control groups/
22	quasi-experiment*.tw.
23	(pre test or pretest or pre-intervention or post test or posttest or post-intervention).tw.
24	exp cohort studies/ or exp Evaluation Studies/ or exp Program Evaluation/
25	(time adj series).tw.
26	Controlled before-after studies/
27	“before and after studies”.tw.
28	(pre test or pretest or pre-intervention or post test or posttest or post-intervention).tw.
29	((intervention or interventional or process or program) adj5 (evaluat* or effect* or outcome*)).tw.
30	((evaluat* or intervention* or treatment*) and (control* or program* or comparison or comparative)).tw.
31	(study or studies).mp.
32	((quantitative or qualitative or mixed method* or cross-sectional) adj2 (design* or approach* or analys?s)).tw.
**33**	**or/14-32**
34	**10 and 13**
35	**33and 34**
