# Supplementary material for: The impact of cash transfers on social determinants of health and health inequalities in Sub-Saharan Africa: a systematic review protocol
Source: Syst Rev. 2016 Jul 13;5:114. doi: 10.1186/s13643-016-0295-4 (PMC4944314; doi:10.1186/s13643-016-0295-4)
Supplement: Additional file 3: — Data extraction form—quantitative studies. (PDF 572 kb) [file 13643_2016_295_MOESM3_ESM.pdf]

Additional file 2: Data Extraction Form – Quantitative studies (Adapted from CPHG, 2011)

**Study ID:**	**Report ID :**	Date form completed:
First author:	Year of study:	Data extractor:
Citation:		

1. General Information

Publication type Journal Article [ ] Working paper[ ] Other (specify e.g. book chapter)________________<br>Report [ ] Conference presentation [ ] Dissertation [ ] Draft report [ ]	
Country of study:	
Funding source of study:<br>Public institution [ ] Private institution [ ]<br>Multilateral Organisation [ ] Government [ ]<br>Not clear [ ] N/A [ ]	Potential conflict of interest from funding? Y / N / unclear<br><br>Is it an independent evaluation (not funded by the implementing agency)? Y / N / unclear
Is there a potential conflict of interest associated with study which could influence results collected/reported?<br><br>If yes, comment	

2. Study Eligibility

**Study Characteristics**			**Page**
**Type of study**	Randomised Controlled Trial (RCT)<br>Cluster Randomised Controlled Trial (c-RCT)	Controlled Before and After (CBA) study Contemporaneous data collection Comparable control site	
	Interrupted Time Series (ITS)<br>Other	Regression discontinuity designs (RDDs)<br>Cross-sectional studies with pre-and post-test measures of the outcomes	
	Does the study design meet the criteria for inclusion?<br>Yes ☐ No ☐ →**Exclude** Unclear ☐		
	Description in text:		

**Participants**	Describe the participants included:		
	Are participants defined as a group having specific vulnerable characteristics?	Yes [ ] No [ ] Unclear [ ]<br>Details:	
	How is the geographic boundary defined?	Details:<br>Specific location (e.g. state / country):	

	Do the participants meet the criteria for inclusion?	Yes [ ] No [ ]→**Exclude** Unclear [ ]	

**Types of intervention**	Intervention consists of direct cash transfers made to households or individuals?	Yes [ ] No [ ] →**Exclude** Unclear [ ]	
	Does the intervention aim to reduce poverty or vulnerabilities?	Yes [ ] No [ ] →**Exclude** Unclear [ ]	
	Type of CT programme	UCT [ ] CCT [ ] Both UCT&CCT [ ] Unclear [ ]	
	If CCT? State the conditions		
	Target beneficiaries of intervention		
	Targeting mechanism of CT		
	Cash transfer size		
	Frequency of cash transfer		
	Indicate any complementary services		
	What is the geographic context of the intervention?	Urban [ ] Rural [ ]<br>[ ] National representative<br>[ ] Other (specify)……………………….	
	State the name of the CT programme		
	State the name of the implementing agency.		
	State the name of intervention funding agency		
	What is the duration of the intervention?		
	Does the intervention meet the criteria for inclusion?	Yes [ ] No [ ] →**Exclude** Unclear [ ]	
**Types of outcome measures**	List outcomes:		
	Outcome measured at a population level or individual level?	Details:	
	Do the outcome measures relate to SDoH?	Yes [ ] No [ ] → **Exclude** Unclear [ ]	

Summary of Assessment for Inclusion

**Include in review** [ ]	**Exclude from review** [ ]
Independently assessed, and then compared? Yes [ ] No [ ]	Differences resolved Yes [ ] No [ ]
Request further details? Yes [ ] No [ ]	Contact details of authors:
**Notes:**	

DO NOT PROCEED IF PAPER EXCLUDED FROM REVIEW

3. Study details

Study intention	Descriptions as stated in the report/paper	Page
Aim of intervention		
Aim of study		
Does this study focus on equity?	[ ] Yes, sub-group analyses are available<br>[ ] Yes, programme is targeted<br>[ ] Yes, programme is targeted & sub-group analyses are available<br>[ ] No, no equity focus<br>[ ] N/A	
Start and end date of the study		
Total study duration		
List countries the study was conducted		

Methods	Descriptions as stated in the report/paper	Page
Method/s of recruitment of participants<br>*(How were potential participants approached and invited to participate?*		
Inclusion/exclusion criteria for participation in study		
Total number of intervention groups		
What is the sample size of the study?		
What is the sample population?		
Representativeness of sample: Are participants in the study likely to be representative of the target population?		
Sample size calculation:<br>What assumptions were made?<br>Were these assumptions appropriate?	(Yes/No/Unclear)	
Was any data imputed?	1. [ ] Yes<br>2. [ ] No<br>3. [ ] Not stated/ unclear	
Was baseline data collected?	1. [ ] Yes<br>2. [ ] No<br>3. [ ] Not stated/ unclear	
Was follow-up data collected?	1. [ ] Yes<br>2. [ ] No<br>3. [ ] Not stated/ unclear	
Was panel data collected?	1. [ ] Yes<br>2. [ ] No<br>3. [ ] Not stated/ unclear	
Was cross-sectional data collected?	1. [ ] Yes<br>2. [ ] No<br>3. [ ] Not stated/ unclear	
Was repeated cross-sectional data collected?	1. [ ] Yes<br>2. [ ] No<br>3. [ ] Not stated/ unclear	
Any comments on data collection/ data type?		

Describe methods of data collection		
What is the frequency of outcome data collection?		
Start date of collection of data on outcome		
End date of collection of data on outcome		

What was the unit of randomisation?<br>Allocation by individuals or cluster/groups<br>(for RCTs & c-RCTs only)		
What was the unit of analysis?<br>Is this the same as the unit of randomisation?	*(Yes/No/Unclear)*	
Statistical methods used and appropriateness of these methods		
Which methods are used to control for selection bias and confounding?	PSM [ ] Covariate matching [ ] DID [ ] IV-regression [ ] Heckman selection model [ ] Fixed effects regression [ ] Other regression [ ] Other [ ]	
**Moderators used in study**	**Descriptions as stated in the report/paper**	Page
Does the study provide information relating to how or why the intervention was effective or not?	Yes [ ] No [ ]	
Was there an analysis of the moderator variable?	Yes [ ] No [ ]	
Indicate the moderator variables used in the study		
What impact do moderators have on effect?		
**Risk of bias**	**Descriptions as stated in the report/paper**	Page
Provide details on the treatment and control group selection (sequence generation: e.g. lottery)		
Is discussion of treatment and control comparability given?	Yes [ ] No [ ] N/A [ ]	
Does the study state variables on which comparability of treatment and control is assessed?	Yes [ ] No [ ] N/A [ ]	
Indicate variables considered in assessment of similarity (e.g. location, poverty levels, vulnerabilities)		
Are covariates in treatment and control groups assessed as balanced, and if unbalanced controlled in adjusted analysis?	Yes [ ] No [ ] N/A [ ]	
State techniques used to match (including matching variables)		
Control is of adequate comparability, moderate adequacy, or not adequate	1 [ ] yes, control is adequate, either through randomisation of selection to intervention and control, or matching, or adjustment regression analysis, or comparability of characteristics which are reported on and are sufficiently similar<br>2 [ ] Adequacy of control is moderate; general statements made on similarity of some variables between treatment and adjustment for confounders in multivariate analysis<br><br>3 [ ] Control is inadequate; nothing reported on similarities between treatment and control groups	
Is control group geographically separated from treatment, or if not separated is it unlikely that comparisons received the intervention?	Yes [ ] No [ ] Not clear [ ] N/A [ ]	
If yes, how do authors control for contamination?		
Was there blinding of participants?	Yes [ ] No [ ] Not clear [ ] N/A [ ]	
Was there blinding of outcome assessors?	Yes [ ] No [ ] Not clear [ ] N/A [ ]	
Was there blinding of data analysts?	Yes [ ] No [ ] Not clear [ ] N/A [ ]	
Describe method(s) used to blind		

**Effect size data**	**Descriptions as stated in the report/paper**	Page
Sample size unit of analysis	Children [ ] Households [ ] Youth/Adolescent [ ] Adults [ ] Other [ ] Not clear [ ]	
Initial sample size treatment group		
Initial sample size control group		
For cluster trials, number of clusters, number of people per cluster		
Number and reason for (and socio-demographic differences of) withdrawals and exclusions for each intervention group		
Were people who entered the study adequately accounted for?		
Number of treatment observations after attrition (individuals)		
Number of control observations after attrition (individuals)		
What treatment effect is estimated?	ITT [ ] ATE [ ] LATE [ ]	
Were there any significant baseline imbalances?	Yes [ ]<br>No [ ]<br>Unclear [ ]<br>Details:	
Control/comparison (what information is provided about what the control or comparison group received?)		

Outcomes

**Question**	**Outcome 1**	**Page/ Para/ Figure**	**Outcome 2**	**Pag e/ Par**
Is there an analytic framework applied (e.g. logic model, conceptual framework)?				
Type of outcome: Is this a modifiable variable (Community level, neighbourhood level, individual level) or desired health outcome				
Time points measured				
Time points reported				
Is there adequate latency for the outcome to be observed?				
Unit of measurement (if relevant)				

How is the measure applied? Telephone survey, mail survey, in person by trained assessor, routinely collected data, other				
How is the outcome reported? Self or study assessor				
Is this outcome/tool validated?				
…And has it been used as validated?				
Is it a reliable outcome measure?				
Is there adequate power for this outcome?				
Were PROGRESS categories analysed by outcome? Indicate the letters of those that outcomes were analysed by (place of residence, race, occupation, gender, religion, education, SES, social capital)				

Results for RCTs/c-RCTS: Dichotomous outcome

Does the study give a precise definition of outcome it assess?			Yes [ ] No [ ] Partially [ ]		
What definition of the outcome is given?					
Comparison					
Outcome					
Subgroup					
Timepoint					
**Results**	**Intervention**		**Comparison**		
	Events	No. participants	Events	No. participants	
State sample size at baseline					
State sample size for treatment group post intervention					
No. of missing participants and reasons					
State result of baseline outcome					
State proportion with outcome at baseline					

State result of post intervention outcome			
State proportion with outcome post intervention (last follow up)			
State result of 1st follow up outcome			
Repeat the above for any additional follow up measures			
Does the study conduct sub group analysis	Yes [ ] No [ ]		
State any sub-groups for which the study includes outcome measures			
Extract data necessary to calculate effect sizes for each outcome where sub-group			
Any other results reported			

For RCT/c-RCT
Continuous outcome

							page/para/fig
Does the study give a precise definition of outcome it assess?			Yes [ ] No [ ] Partially [ ]				
What definition of the outcome is given?							
Comparison							
Outcome							
Subgroup							
Timepoint							
Post-intervention or change from baseline?							
**Results**	**Intervention**			**Comparison**			
	Mean	SD (or other variance)	No. participants	Mean	SD (or other variance)	No. participants	
State result of baseline outcome							
State result of post intervention outcome							
State result of 1st follow up outcome measure							
Repeat the above for any additional follow up measures							

No. missing participants and reasons							
Any other results reported							

For CBA

Page/para/fig

Comparison			
Assignment	How were control and treatment groups selected?? Is there likely to be an effect if these were the opposite way?		
	Contemporaneous data collection?		
Outcome			
Subgroup			
Timepoint			
Post-intervention or change from baseline?			
	**Intervention**	**Comparison**	
No. participants measured			

No. missing participants and reasons			
Baseline result (with variance measure)			
Post-intervention results (with variance measure)			
Change (Post – baseline) (with variance measure)			
Difference in change (intervention – control) (with variance measure)			
Any other results reported			

For ITS

			Page/para/fig
Comparison			
Outcome			
Subgroup			
Length of timepoints measured			
Snapshot or interval measured			
No. participants measured			
No. missing participants and reasons			
	Pre-intervention	Post-intervention	
No. of timepoints measured			

Mean value (with variance measure)			
Difference in means (post – pre)			
Percent relative change			
Result reported by authors (with variance measure)			

Other relevant information

Were outcomes relating to unintended effects of the intervention described? Include any data for these in the outcomes tables above		
What are the barriers/facilitators of the success of the CT intervention?		
Do the authors describe any political or organisational context?		
Was a process evaluation conducted?		
Potential for author conflict *ie. evidence that author or data collectors would benefit if results favoured the intervention under study or the control*		

Key conclusions of the study authors		
Could the inclusion of this study potentially bias the generalisability of the review? Equity pointer: Remember to consider whether disadvantaged populations may have been excluded from the study.		
Is there potential for differences in relative effects between advantaged and disadvantaged populations?		
References to other relevant studies		
Additional notes by review authors		
Correspondence required for further study information (from whom, what and when)		
