# Supplementary material for: The impact of cash transfers on social determinants of health and health inequalities in Sub-Saharan Africa: a systematic review protocol
Source: Syst Rev. 2016 Jul 13;5:114. doi: 10.1186/s13643-016-0295-4 (PMC4944314; doi:10.1186/s13643-016-0295-4)
Supplement: Additional file 4: — Data extraction form—qualitative studies. (PDF 536 kb) [file 13643_2016_295_MOESM4_ESM.pdf]

Additional file 3: Data Extraction Form – Qualitative studies (Adapted from JBI, 2011)

**Study ID:**	**Report ID :**	Date form completed:
First author:	Year of study:	Data extractor:
Citation:		

1. General Information

Publication type Journal Article [ ] Working paper[ ] Other (specify e.g. book chapter)________________ Report [ ] Conference presentation [ ] Dissertation [ ] Draft report [ ]	
Country of study:	
Funding source of study:<br>Public institution [ ] Private institution [ ]<br>Multilateral Organisation [ ] Government [ ]<br>Not clear [ ] N/A [ ]	Potential conflict of interest from funding? Y / N / unclear<br><br>Is it an independent evaluation (not funded by the implementing agency)? Y / N / unclear
Is there a potential conflict of interest associated with study which could influence results collected/reported?<br><br>If yes, comment	

2. Study methodology

**Study Characteristics**			**Page/ Para/ Figure #**
**Study methodology & Type**	☐ Phenomenology<br>☐ Interpretive	☐ Descriptive	
	☐ Social constructionism	☐ Other	
	☐ A process evaluation	*Is the study design clearly started?*<br>Yes ☐ No ☐ Unclear ☐	
	Is it a stand-alone study?<br>Yes ☐ No ☐ Unclear ☐	Is it conducted alongside quantitative study? Yes ☐ No ☐ Unclear ☐	
	Description in text:		

**Participants**	Describe the participants included:	

	Are participants defined as a group having specific vulnerable characteristics?	Yes [ ] No [ ] Unclear [ ]<br>Details:	
	How is the geographic boundary defined? (urban, rural)		
	Socio-demographics (e.g. age, sex, religion, occupation, etc.)		
	Do the participants meet the criteria for inclusion?	Yes [ ] No [ ]→**Exclude** Unclear [ ]	

**Types of intervention**	Intervention of consists of direct cash transfers made to households or individuals?	Yes [ ] No [ ] →**Exclude** Unclear [ ]	
	Does the intervention aim to reduce poverty or vulnerabilities?	Yes [ ] No [ ] →**Exclude** Unclear [ ]	
	Type of CT programme	UCT [ ] CCT [ ] Unclear [ ]	
	If CCT? State the conditions		
	Target beneficiaries of intervention		
	Cash transfer size		
	Frequency of cash transfer		
	Indicate any complementary services		
	What is the geographic context of the intervention?	Urban [ ] Rural [ ]	
	What is the cultural context of the intervention?		
	Does the intervention meet the criteria for inclusion?	Yes [ ] No [ ] →**Exclude** Unclear [ ]	
**Phenomena of interest**	List phenomenon of interest (e.g. beneficiaries lived experience of CTs, social capital, perceived programme impact, process evaluation of CTs barriers and facilitators, etc.)		
	Do the phenomena of interest meet the criteria for inclusion?	Yes [ ] No [ ] → **Exclude** Unclear [ ]	

Summary of Assessment for Inclusion

**Include in review** [ ]	**Exclude from review** [ ]
Independently assessed, and then compared? Yes [ ] No [ ]	Differences resolved Yes [ ] No [ ]
Request further details? Yes [ ] No [ ]	Contact details of authors:
**Notes:**	

DO NOT PROCEED IF PAPER IS EXCLUDED FROM REVIEW

3. Study details

Study intention	Descriptions as stated in the report/paper	Page
Aim of intervention		
Aim of study		

Methods & Results	Descriptions as stated in the report/paper	Page
Ethics – how ethical issues were addressed		
Any theoretical/conceptual framework used in the study?		
Method/s of recruitment of participants *(How were potential participants approached and invited to participate? Where were participants recruited from?*)		
Indicate the sampling approach		
Inclusion/exclusion criteria for participation in study		
Data collection methods		
Data analysis techniques		
Themes emanating from data		
Data extracts related to the key themes		
Key study findings/results		
Authors explanations		
Authors conclusions		
References to other relevant studies		
Additional notes by review authors		
Correspondence required for further study information (from whom, what and when)		
