# Supplementary material for: The impact of cash transfers on social determinants of health and health inequalities in Sub-Saharan Africa: a systematic review protocol
Source: Syst Rev. 2016 Jul 13;5:114. doi: 10.1186/s13643-016-0295-4 (PMC4944314; doi:10.1186/s13643-016-0295-4)
Supplement: Additional file 5: — Risk of bias assessment. (PDF 158 kb) [file 13643_2016_295_MOESM5_ESM.pdf]

Additional file 2: Risk of bias assessment: RCTs, CCTs, CBAs & ITS

Domain	Yes	No	unclear	Description	Ref. page
Was the allocation sequence adequately generated?					
Was allocation adequately concealed?					
Were baseline outcome measurements similar?					
Were baseline characteristics similar?					
Was knowledge of the allocated intervention adequately prevented during the study (blinding of participants and personnel)?					
Was the study adequately protected against contamination?					
Was there a blinding of outcome assessment?					
Were incomplete outcome data adequately addressed (attrition bias)?					
Are reports of the study free of suggestion of selective outcome reporting (reporting bias)?					
Was the study free from other risks of bias?					
**ITS**					
Was the intervention independent of other changes?					
Was the shape of the intervention effect pre-specified?					
Was the intervention unlikely to affect data collection?					
Was knowledge of the allocated interventions adequately prevented during the study?					
Were incomplete outcome data adequately addressed?					
Was the study free from selective outcome reporting?					
Was the study free from other risks of bias?					

* Note: For each section above ‘Yes’ indicates a ‘low risk of bias’; ‘No’ indicates a ‘high risk of bias’; ‘Unclear’ indicates an ‘uncertain risk of bias’.
