# Supplementary material for: The impact of cash transfers on social determinants of health and health inequalities in Sub-Saharan Africa: a systematic review protocol
Source: Syst Rev. 2016 Jul 13;5:114. doi: 10.1186/s13643-016-0295-4 (PMC4944314; doi:10.1186/s13643-016-0295-4)
Supplement: Additional file 6: — Critical appraisal of qualitative studies. (PDF 150 kb) [file 13643_2016_295_MOESM6_ESM.pdf]

Additional file 3: Critical Appraisal Checklist for Qualitative Studies (Adapted from JBI, 2011)

Reviewer…………………………………………Date………………………….

Author……………………………………………Year……………………..Record #..................

1. Is there congruity between the stated philosophical Perspective and the research methodology? Yes[ ] No [ ] Unclear [ ] N/A [ ]

2. Is there congruity between the research methodology and the research question or objectives? Yes[ ] No [ ] Unclear [ ] N/A [ ]

3. Is there congruity between the research methodology and the methods used to collect data? Yes[ ] No [ ] Unclear [ ] N/A [ ]

4. Is there congruity between the research methodology and the representation and analysis of data? Yes[ ] No [ ] Unclear [ ] N/A [ ]

5. Is there congruity between the research methodology and the interpretation of results? Yes[ ] No [ ] Unclear [ ] N/A [ ]

6. Is there a statement locating the researcher culturally or theoretically? Yes[ ] No [ ] Unclear [ ] N/A [ ]

7. Is the influence of the researcher on the research, and vice-versa, addressed? Yes[ ] No [ ] Unclear [ ] N/A [ ]

8. Are participants, and their voices, adequately represented? Yes[ ] No [ ] Unclear [ ] N/A [ ]

9. Is the research ethical according to current criteria or, for recent studies, and is there evidence of ethical approval by an appropriate body? Yes[ ] No [ ] Unclear [ ] N/A [ ]

10. Do the conclusions drawn in the research report flow from the analysis, or interpretation, of the data? Yes[ ] No [ ] Unclear [ ] N/A [ ]

*Overall ratings: High [ ] Moderate [ ] Low [ ] Very low [ ]

Comments:…………………………………………………………………………………………
…………………………………………………………………………………………………
…………………………………………………………………………………………………
…………………………………………………………………………………………………
…………………………………………………………………………………………………
……………………………………………………………………………………………….
